# Changes in professionals’ beliefs following a palliative care implementation programme at a surgical department: a qualitative evaluation

**DOI:** 10.1186/s12904-017-0262-4

**Published:** 2017-12-28

**Authors:** Pia Hahne, Staffan Lundström, Helena Leveälahti, Janet Winnhed, Joakim Öhlén

**Affiliations:** 1 Stockholms Sjukhem Foundation, Mariebergsgatan 22, 112 35 Stockholm, Sweden; 2 Ersta Sköndal Bräcke University College, Box 111 89, -100 61 Stockholm, SE Sweden; 3 Department of Oncology-Pathology Karolinska Institutet, Stockholm, Sweden; 4 ASIH Praktikertjänst Västerort N.Ä.R.A, Vällingby, Sweden; 5 Institute of Health and Care Sciences and University of Gothenburg Center for Person-Centered Care, Salgrenska Academy at the University of Gothenburg, Gothenburg, Sweden

**Keywords:** Focus groups, General surgery, Health plan implementation, Palliative care, Palliative medicine, Patient care team, Qualitative research

## Abstract

**Background:**

One ambition regarding palliative care is that it should be more accessible to patients and families regardless of care setting. Previous studies show many difficulties and shortcomings in the care of patients with palliative care needs in acute care facilities, but also challenges regarding efforts to implement palliative care. The aim of this study is to evaluate how the implementation of palliative care, using a combination of integration and consultation strategies, can change beliefs regarding palliative care among professionals in a surgical department.

**Method:**

In order to explore professionals’ experiential outcome of an educational implementation strategy, a before-after qualitative design was used. The study was based on three focus group discussions. Two discussions were conducted before introducing the implementation strategy and one was conducted after. The participants consisted of five nurses and two specialist surgeons from a surgical department in Sweden. The focus group discussions revealed a variety of different attitudes and beliefs, which were analysed using qualitative systematic text condensation.

**Results:**

Beliefs regarding palliative care were identified in seven areas; the importance of palliative care, working methods in palliative care, team collaboration in palliative care, collegial support, discussions about diagnosis, symptoms at the end of life, and families of patients in palliative care. Changes in beliefs were seen in all areas except one: team collaboration in palliative care.

**Conclusion:**

It is possible to change the beliefs of health care professionals in a surgical department regarding palliative care through the implementation of palliative knowledge. Beliefs were changed from an individual to a collective development where the group initiated a shared palliative working method. The changes observed were palliative care being described as more complex and participants differentiating between surgical care and palliative care.

**Electronic supplementary material:**

The online version of this article (10.1186/s12904-017-0262-4) contains supplementary material, which is available to authorized users.

## Background

The basic philosophy of palliative care is to relieve symptoms and enable the best possible quality of life for patients and their families when cure is no longer possible and achieving this through a multidimensional and team oriented approach. The goal is to neither prolong nor shorten life [1]. One ambition is that patients and their families should have increased access to palliative care in all facilities where care is provided. The literature emphasises that it is the interdisciplinary teams that are able to provide fundamental knowledge regarding palliative care [2]. This is important considering access to early palliative care can result in an improved quality of life and longer survival time for patients with palliative care needs [3]. However, studies show that health care professionals lack education in palliative care, and that the knowledge they do have in this area has usually been acquired through clinical experience [4–8]. Poor communication concerning patients with palliative care needs and teams not working together towards the same goal have also been described [9]. Education in palliative care can therefore be considered as something that should be given to all members of the health care team [4] and the principles of palliative care should be integrated into the daily practice of all health care professionals [7].

Palliative care has been implemented in many different ways in a variety of care settings with the aim of giving support to patients, their families, health care professionals and health services, but with mixed results. Some attempts at implementation have highlighted that clinically, there is confusion and ambiguity regarding the concept of palliative care [10, 11]. This has created difficulties in determining which patients should receive palliative care, who should provide palliative care and when the care should transition to a palliative phase, which results in an inconsistency in the care [12] that patients receive [10]. According to Pronovost et al. [13] a difficult challenge in the integration of palliative care can be the actions taken by health care professionals, since resuscitation is an automatic response in acute care [8]. In addition, due to the structures of work in health care, the care of a patient may involve several consulting physicians and there may be frequent rotation of health care staff [14].

The implementation of palliative care into acute care, especially in surgical practice is often characterised as breaking with the accepted beliefs (conscious or unconscious) that the goal of all patients is to survive [9, 15] and that health care professionals have given up hope and do nothing more for the patient [12]. For that reason surgical care could be linked to beliefs that differ from palliative care. Recently, although less frequently occurring, a new approach is starting to be adopted where palliative care is given alongside other treatment, irrespective of the patient’s prognosis [8]. However the evidence for implementation of palliative care in the context of surgical care is sparse and there is no support for choosing a special approach [16], but to focus on communication, decision making and delivery of palliative care to improve patient outcomes with an overall goal to improve patient’s well-being and quality of life [17].

Like many people, health care professionals have particular attitudes and beliefs and adopt particular working methods without necessarily understanding the basis for them. This may result in their actions being something other than those intended [18]. According to Benner [19] health care professionals can form common beliefs through contact with patients and their families and learning the different implications of patterns of reaction to and possible courses of action in extreme situations. With time, these beliefs create an approach, a tradition that can develop into common overall patterns and a tendency to act in a particular way in a given situation.

The integration of palliative care into health care is predominantly described as being based on a consultation strategy or an integration strategy [7, 8, 20]. The consultation strategies focuses on increasing involvement and effectiveness with regard to the consultation of specialist palliative care teams. Checklists are sometimes used to initiate such a consultation. The integration strategy aims to embed palliative care principles and interventions into daily practice. This approach implies joint discussions between the acute care team and the palliative care team so that suggestions for common strategies can develop into reciprocal learning [7, 8, 20]. A combined approach incorporates elements from both strategies, which can possibly be seen as the most successful approach [8]. Hua et al. [21] argue that a combination of both strategies is necessary to be able to address the palliative care needs of seriously ill patients. Further, implementation science frameworks can be used to guide the implementation process. PARiHS (Promoting Action on Research Implementation) is a practical tool used not only to guide implementation, but also to understand in an analytical way the essential factors and their relation to each other in the implementation of research findings. According to the PARiHS model, the three most important factors in implementation are *evidence, context* and *facilitation* [22].

The goal should be to bring about an interaction between previous experience from acute care, palliative care expertise and research in order to facilitate implementation [22]. The belief that death means failure needs to be changed to regarding death as a natural part of life that is worth acknowledging and preparing for [23]. According to McCormack et al. [24] the key elements for successful implementation are based on culture, leadership and methods of evaluation. This requires all disciplines in the team to support a culture where everyone regards palliative care as an integral part of standard acute care [7]. Successful implementation needs a supervisor who can understand the evidence and make it comprehensible and relevant, and who supports the process of change [25]. With an increasing need for, and interest in the implementation of palliative care into acute care [16, 17] it is important to evaluate if a strategy for implementation of palliative care can change the beliefs of professionals in a surgical department with regard to palliative care. For this reason the aim was to evaluate how the implementation of palliative care, using a combination of integration and consultation strategies, can change beliefs with regard to palliative care among professionals in a surgical department.

## Methods

### Design

In order to explore the experiential outcome of an educational implementation strategy, a before-after qualitative design was used [26]. For evaluative purposes the analyses were performed by two researchers (1st and last authors) who were not involved in the implementation process or the data collection, while three researchers (2nd, 3rd and 4th authors) were primarily involved in the implementation process and/or the data collection.

### Implementation strategy

To enhance sustainable implementation of palliative care at a surgical department, a combination of integration and consultation strategies [7, 8, 20] was used for the implementation. For the integration strategy, a team was formed consisting of five nurses and two specialist surgeons from three wards in a surgical department. This team received education at the surgical department from palliative care specialists comprising one consultant, one registered nurse and one clinical nurse specialist. The education consisted of 12 seminars each lasting 120 min; the opportunity for reflection and discussion was provided throughout the whole of the project which lasted one year. The seminars were based on actual patient cases from the surgical department and were presented by the participants. Case discussions were mixed with short in-depth theoretical education which was relevant to the specific cases and covered the following: symptom relief – pertaining pain, nausea, fatigue, anxiety/confusion, cachexia and nutrition; dying; communication with focus on feedback; educational approach and the support of families. The participants were sent the cases in advance of each seminar and were also given literature on palliative care: each participant received the National Clinical Practice Guidelines for palliative care [27] and the three participating wards were given study literature in the form of a textbook about palliative medicine and care [28]. The structure of the seminars with case discussions, theoretical education and reflection was maintained throughout the project period. On two occasions during the project, the participants were asked to evaluate the seminars and case discussions in order to give the palliative care specialists the opportunity to develop and improve upcoming case discussions. The additional consultation strategy consisted of twice weekly consultative visits to the three surgical wards by a consultant physician in palliative medicine which were made throughout the project. In discussion with the surgeons, a palliative assessment of a total of 82 inpatients was carried out during ward rounds.

### Sample

Since a specialized palliative care and an acute care hospital (both private; one non-profit and one for-profitmaking) both had a common interest in improving the care of the most seriously ill surgical patients, a surgical department with three wards in the capital city of Sweden was chosen for the study. All members of staff employed at the surgical department showing inters were invited to participate and all who volunteered were offered to participate. Thus, the sampling of participants emanated from the participants personal interest and/or through personal contacts. The participants consisted of nurses and surgeons with varied experience in their respective professions (Table 1). The age range of the participants was approximately 25–60 years. An initiator surgeon and that the two professionals, nurses and surgeons were the key groups on the surgical department were the reason for the sample chose.

**Table 1:** Participants in the 3 focus group discussions

	Discussion 1	Discussion 2	Discussion 3
	January 2013	February 2013	January 2014
Participant	Two women	Two women	Five women
	One man	One man	One man
Participant profession	Two nurses	Two nurses	Five nurses
	One surgeon	One surgeon	One surgeon
Interviewer	Two women	Two women	One man
	One man	One man	
Interviewer profession	One consultant physician PhD	One consultant physician PhD	Consultant physician
	One registered nurse with Licentiate degree	One registered nurse with Licentiate degree	
	One registered nurse	One registered nurse	
Interviewer during the discussion	One 1st mentor	One 1st mentor	One mentor
	One 2nd mentor	One 2nd mentor	
	One observer	One observer	

### Data collection

Focus group discussions were held with the team receiving education before and on completion of the project to evaluate if the education programme had affected participants’ beliefs regarding palliative care. The focus group discussions were audio recorded. The project was carried out over a period of one year where two focus group discussions were conducted at the start of the project due to difficulties to get all the participant together at the same time and a final focus group discussion was held one year later.

The aim of the three focus group discussions was to generate discussion about palliative care. The goal was not to reach consensus on the issues discussed nor to find solutions to these, but instead to obtain different views regarding these issues. An additional goal was for the discourse on palliative care to be as open as possible in order to reveal variations in attitudes and beliefs [29].

One participant withdrew participation in the middle of the project and another participant, who had not been present from the start of the project, took part in the third and final focus group discussion. In the final focus group discussion the first mentors and observer were replaced by a new independent mentor in an attempt to minimise the possibility that experience gained by the mentors during the project could influence the discussions. The new mentor had experience of focus group discussions and had not been involved in the project or in the earlier focus group discussions.

The focus group discussions were based on 13 issues focusing on various aspects of core concepts in palliative care: symptoms, communication, team collaboration, and family [28]. During each focus group discussion the mentor introduced the subjects for discussion and ensured an exchange of ideas by creating a climate that allowed participants to express their own personal views [29]. Follow up questions were posed when there was a need for clarification.

### Analysis

The process used for analysis is based on Malterud’s [26] systematic text condensation, an analysis strategy developed from traditions common to most methods that are used for descriptive analysis of qualitative data. Malterud’s method offers the researcher a process of intersubjectivity, reflexivity and feasibility.

The analysis was carried out in four steps [30].

In the first step, *total impression – from text to themes*, the three transcribed discussions were listened to and read through in order to get an overall impression of the material. The first two focus group discussions conducted before implementation were then analysed. A summary and a first organisation of data based on material from these discussions resulted in three broad themes: palliative care, health care professionals, and family.

In the second step, *meaning units – from themes to codes*, text that in some way or another contained information relating to the themes from the first step, so called *meaning units*, was sorted and coded according to the themes from the first step.

In the third step, *condensation – from code to meaning*, the meaning units were sorted into code groups. The third focus group discussion, conducted after the implementation programme, was then analyzed in a way similar to the analysis of the two previous discussions. The codes in the third focus group discussion were found to be consistent with the codes from the two discussions conducted before the implementation programme. The material in each respective code group from all three focus group discussions was sorted into corresponding subgroups under the headings “before” and “after” the implementation with the help of a table. Empirical data were reduced to elements with the same meaning, a decontextualised selection.

In the final step, *summarising – from condensation to descriptive meanings*, material before and after implementation was summarised into a common text with seven areas in order to clarify the way in which beliefs regarding palliative care had changed [30].

Rigour and credibility in the analysis was secured in several ways. Attempts were made throughout the whole analysis process to focus on meaningful and distinguishing characteristics in the material. The intention was to maintain a reflective relationship regarding the processing of the data and to recount the participants’ experiences as accurately as possible. Efforts were made to maintain a responsible level of methodological stringency [26]. The nurse (the 4th author, observer), who was present at the first two focus group interviews, scrutinised the summary and confirmed that the results were consistent with her experience of the focus group discussions [29]. Alternative interpretations we discussed within the research team and in seminar discussions with colleagues led to, at times to re-analysis to confirm, reject and refine the tentative interpretation. In this way, we claim, it was a transparent process where we shared and processed thoughts before interpretations were finalized. The participants’ and the palliative care specialists’ reflections regarding participation in the implementation are presented in Additional file 1.

## Results

Beliefs regarding palliative care were identified in seven areas: the importance of palliative care, working methods in palliative care, team collaboration in palliative care, collegial support, discussions about diagnosis, symptoms at the end of life, and family of patients in palliative care. Changes in beliefs were seen in all areas except one, team collaboration in palliative care.

### The importance of palliative care

In discussions prior to implementation, the participants expressed fairly general beliefs about ​​what palliative care involves, beliefs that to some extent were reminiscent of how palliative care is described in the literature, but also beliefs that reflected a degree of uncertainty.

1K1: *Yes, it is if you get some kind of ...experienced ... yes, well-being about something ... so yes ....*

1H: *On some level*.

1K1: *Quality of life or whatever you should say, feeling as little ill as possible maybe. It's difficult ... you want to achieve a sort of well-being, for them and their relatives.*

1M: *I agree. I think it’s difficult to be more specific than that. It’s to achieve the absolute best level of quality of life for the patient ... It involves an awful lot .....*

After implementation, a change in the participants’ earlier beliefs could be seen when they described the differences between the two types of care.

3K3: ... ..*it is something quite different from this acute care, and you have more time and it seems to be a bit more dignified in many cases ...*

However, participants also described beliefs that patients needing palliative care endure a great deal of suffering that is difficult to relieve, and that not everything can be cured even if optimal care and good symptom relief are given.

Before the implementation there was a view that patients with palliative care needs were not considered as being as acute as the other patients on a surgical ward. The belief was that the wish of all patients is to be able to live as long as possible. The participants felt that there is always an element of palliative care on a surgical ward, but there was no routine for determining whether a patient was considered to be in need of palliative care or not. Participants related these beliefs to lack of knowledge, but that other obstacles such as a difference of opinions between colleagues were also present.

There was a belief that everything needed to be more formalised in order to be able to introduce all the new knowledge to colleagues who had not been involved in the project. But there was still a slight worry regarding differences of opinions, which was seen from the participants’ beliefs about how the new knowledge would be received.

3R: *But this is a colossus that has to be moved, it's not enough that we can think this way, it is all the staff that will have to do it.*

### Working methods in palliative care

The focus group discussions conducted before the implementation revealed that working methods were based on each participant having formed their own beliefs about palliative care according to how they themselves would like it, and/or previous experience. Initially, these beliefs were mostly described in the first person; *I usually do, I think, for my part ...* but after the implementation the participants considered themselves to be more reflective, to then be able to individualise the care. They related this to their increased confidence in how to respond to patients with palliative care needs.

3R: *I have become more inclined to maybe stop and be a bit more selective ... depending on feedback and such ....*

Prior to implementation there was a belief that, in the first meeting with a patient, the focus was most often on acute interventions before any thought was given to the initiation of palliative measures.

2K1: *How can we get the patient to feel well, you know before you maybe start thinking about palliative measures and such.*

The implementation had changed the beliefs to wanting to include palliative care in their work and to give quality of life to patients with palliative care needs during the time the patient was still lively and alert.

The belief before the implementation was that palliative care often involves not having enough time and poor communication, which negatively affected the method of working. According to participants this resulted in lack of continuity towards patients, their families and towards colleagues. This made it difficult for health care professionals to form relationships built on confidence and trust, which also reduced the possibility of influencing the care of the patients.

Knowledge about palliative working methods gained through the implementation resulted in beliefs that continuity increases a sense of security for all patients, not just those in need of palliative care. Lack of patient continuity also made it difficult for the health care professionals to maintain a palliative approach. This resulted in the desire for some form of checklist to be able to know what has already been done and what needs to be done, in order to make the work easier for all involved.

### Team collaboration in palliative care

Before implementation there was a belief that the surgical care team was not synchronised, one colleague did not know what other colleagues were doing, *... I am not aware of ... your input and it bothers me but I don’t get to grips with it, I don’t ask ....* (1 M).

Some surgeons were not happy to work with inexperienced nurses and there was a belief that nursing assistants were sometimes not fully aware of how seriously ill the patients were. After implementation, there was still a belief that there was a lack of collaboration regarding patients in need of palliative care.

3 K3: *Unfortunately I don’t think we work as a team with the patient, it would be much easier if nursing assistants, physicians and nurses all worked together and with the relatives too.*

It was believed that the patient had contact with many different teams. Nevertheless, implementation had created beliefs that a functioning team could provide greater collective knowledge about the patient, which would make the work easier.

### Collegial support

It was generally believed by the participants prior to the implementation that they gave each other support when needed. There were no organised times in the daily routine for discussion or feedback.

1K1: *I think that we back each other up fairly well if someone makes it known that they think something is difficult ... there is no-one who says yeah, you’ll have to take care of that yourself*

After implementation, there were still no organised times for discussion; it was believed that discussion and feedback consisted of debriefing during coffee breaks where not all the parties concerned were present.

Before the implementation it was believed that the surgeons turned to their medical colleagues since they were more familiar with that role, while nurses and nursing assistants turned to each other. When feedback was given between professional groups it was usually the nurse who gave feedback to the surgeon. Reasons for this was that the surgeons believed that their work often involved working alone, and that they did not know much about the nurses’ work. Any feedback given was believed to always involve praise, one reason for this was thought to be fear of how critical feedback would be received. Patients were believed to give more feedback than colleagues.

2K2: ... *But just between colleagues, we are very bad at ... ..it hardly exists.*

The implementation encouraged discussions that resulted in the idea of a surgical culture where you did not always reveal how you felt, or chose not to talk about it. However, it was also believed that the needs for discussion regarding patients on a surgical ward are different since not all the patients are palliative.

### Discussions about diagnosis

Initially it was believed that the task of informing patients was carried out without the involvement of the team; it was considered to be something that the surgeons performed alone. The surgeons were aware that these discussions were influenced by personal views and experiences, and the discussions were believed to vary enormously. Such discussions often ended with a referral being made to someone outside of the medical care, for example a social worker. Participants revealed that the implementation had started to form their own ideas about what a discussion with a patient could be like when the focus of care had become palliative. Although it was still the surgeon who most often gave difficult information alone, there were also ideas about clarifying the structure of such discussions, and having a model to follow.

Previous to the start of team consultation it was believed that the surgeons frequently received more information. Lack of cooperation regarding the sharing of information meant that the nurses on duty did not always know that the surgeon had discussed with the patient.

1K1: ... *it's maybe good that someone who is still on the ward is included, because when you go ... it’s very empty and maybe, as a member of staff, I don’t even know that you had just been there and said what you said ...*

In the last focus group discussion after the implementation period, the participants’ roles regarding sharing of information were seen to have been clarified. Usually the surgeon informed the patient about the diagnosis and the nurse was there to offer support after the discussion. Changes could be seen regarding follow-up discussions when difficult news was given. Before implementation, the belief was that these discussions were conducted by the surgeon, as far as was possible. The nurses said that they tried to talk to patients irrespective of whether they were terminally ill or not. Following the implementation the belief emerged that health care professionals could themselves monitor the psychological process of patients after they had received difficult news; the participants thought that their presence and their way of giving information could give the patient hope.

Both before and after implementation there was frustration in the nursing group that information about diagnosis was given to patients while they were being cared for in a four-bedded room. It was believed that this could have negative consequences for the patient, for other patients and for the nurses’ work.

### Symptoms at the end of life

The symptoms that were believed to be most difficult for patients in need of palliative care preceding the education programme were nausea and pain. The symptoms believed to be most challenging for the surgical care team were pain, nausea and loss of appetite. The symptoms that the participants wanted increased knowledge about were pain, nausea, nutrition and itching, but also lethargy, for example how as health care professionals they can motivate these patients to feel quality of life and a sense of joy. There were also concerns about symptom management.

2K1: *Yes, or for those who get breakthrough pain you can inject so much morphine that they are quite drowsy, completely out of it. That's no fun either having to do that.*

After the implementation, palliative symptoms were described as more complex, for example difficulties with nutrition. The participants also raised the issue of psychological symptoms such as anxiety. In addition, they described beliefs that the way they assessed the patients’ symptoms could affect the symptom profile.

3K2: *You didn’t see it at all from just looking at her, but when you talked to her .....*

Beliefs in a person-centered approach regarding symptoms in connection with palliative care were apparent both before and after implementation, which were expressed as symptoms being seen as different for individual patients and that there were many different factors that could affect the symptoms.

### The families of patients in need of palliative care

It was reported before the implementation that family members were seldom seen as a resource in the palliative care of a patient, but that they could instead indirectly affect the patient negatively. This was characterised by the belief that the attitudes of family members could have a direct impact on the staff.

2M: *It's hard and it wears down more staff than any ... I've really seen how the atmosphere on a ward deteriorates, for it does sometimes with us, gets worse just like that.*

However, there was understanding for the way that family members acted since there was a belief that they could be in different phases of shock. Following the implementation this belief had changed to thinking that the behaviour of the family could be the result of painful reactions to grief and loss. It was believed that these emotions could be difficult for the family to cope with and they needed to take their reactions out on someone.

There was a belief previous to the implementation that family members needed to talk to someone not involved in health care, for example a social worker or a priest. After the implementation the nurses’ beliefs had changed to thinking that the family might wish to talk to the person who works most closely with the patient. As a result of this, the nurses had started offering to do this. They believed that they could help the family to see that what is taking place is real, that what is happening is natural and normal.

Discussions with the family before the implementation were believed to consist of a dialogue, but that the family had no time for reflection and the surgeons very rarely asked them how they were feeling. The implementation raised different ideas for possible improvements to create a more ideal set up for such discussions, and also a model that could be followed. In addition, it was thought that discussions with the family should be made compulsory, but that this might be difficult since not all the patients on the ward were receiving palliative care.

Before the implementation it was believed that when discussions with the family were initiated, they were started casually by a nurse or by the family themselves; surgeons also initiated discussions regarding changes to investigations and/or prognosis. Doubts about initiating discussions were due to beliefs that the family would think that they were about to receive difficult information and that the family was believed to be already aware of the situation. After the implementation it was still thought that arranging discussions with the family could be interpreted as a bad omen. However, there was also the belief that such a discussion could be a good thing even when giving difficult information, and it could save time. The family members were thought to go from speculation and fear to relief, and that uncertainty was more difficult for them to cope with.

*3R: ... it becomes a win-win situation, even with giving sad information, I would say. If you do it well then it is very much better health care for the staff, the patients, the families and everyone will be satisfied ....*

## Discussion

Based on the responses after implementation, the participants’ beliefs had changed regarding the importance of palliative care, working methods in palliative care, collegial support, discussions about diagnosis at the end of life, and the family of patients in palliative care. No changes were shown regarding team collaboration. Common to these changes in beliefs was descriptions of the differences between surgical care and palliative care. After implementation, palliative care was described as more complex and more compound. Before implementation it was believed that the focus was often on acute interventions and that all patients want to be able to live for as long as possible. The belief that palliative care can involve suffering that is difficult to relieve was described after implementation; not everything can be cured, even if optimal care and good symptom relief are available. Beliefs were also described regarding giving patients in need of palliative care good quality of life before it was too late, giving hope that goes beyond cure and survival. In addition, palliative care was believed to be a little more dignified and that it creates more continuity with regard to contact with patients, their families and with colleagues. Before the implementation programme the participants spoke about palliative care as being based on the individual’s own professional perspective, experience and preference; with this in mind, the change in beliefs after implementation can be interpreted as them beginning to develop a collegial understanding of palliative care. Participants stressed that they had gained an increased awareness of their own professional role and the results reflected beliefs about both strengths and weaknesses in how to manage contacts with patients with palliative care needs, which can be assumed to be a start towards developing a common method of working [19]. This is also supported by Friedrichsen et al. [31] who reported that professionals’ perceptions in regard to palliative measures could change following implementation of palliative care specialist consultations; to which our study contributes with a suggestion for how beliefs can change.

One goal with an implementation programme is to develop appropriate and sustainable health care for patients and families. The combined integration and consultation strategies were used to actively disseminate palliative care. The use of a clinical guideline and a text book [27, 28] could be considered a top-down strategy, while sharing case stories and reflections in seminars were more of a bottom-up strategy. The reasoning among the participants’ beliefs showed questioning and critical reflection on previous well-recognized views and habits, and change thereof has in implementation research been conceptualized as creating prerequisites for a development oriented learning. However, this study included no data regarding to what extent the change in beliefs was aligned with behaviour changes of the professionals. Although it is well known that manifest clinical behaviour usually is stable and habitual, the inclusion of palliative care consultations implied a change in the direct clinical context – a palliative medicine consultant regularly visiting, in person, a surgical team and taking part in shared clinical decision-making – and this is a known facilitator for successful implementation [32].The belief changes disclosed could be regarded as specific areas of importance for the contextual importance, which adds to well-known general domains in implementation science [33]. To maintain new knowledge in a working group there is a need for support from coworkers that share similar beliefs otherwise it would may be difficult to retain it [34]. Before beliefs influence behaviour the beliefs have to result in clinical practice, clinical practice that may develop lasting behaviours, a foundation to build on.

Previous implementation of palliative care has shown positive results [31, 35–37] as was also shown in this study since it was believed that participants felt more confident when their knowledge and their presence seemed to influence patients and their families. However, even after implementation, it was believed that structure and routine were still lacking in the day-to-day work with patients with palliative care needs. According to Wheelan [34] a working group needs clear goals regarding the duties, profile and direction of their work and the group should have a function, tasks and objectives. Due to this perceived lack of structure, participants were also seen to have difficulty in passing on new knowledge to colleagues who had not been involved in the project. The comprehensive use of a framework for the implementation [e.g. 22] might have produced a different result regarding structure since the interaction between the three basic elements of evidence, context and facilitation can be crucial for how successful the implementation of change will be. If any of these factors are lacking then success will not be achieved.

It is also possible that a lack of structure also related to the different beliefs regarding suggestions for improvement that were described in the results as checklists, a model to follow in the following areas: working methods, discussions about diagnosis, and discussions with the family. It was believed that such checklists could facilitate the work in general and provide support during discussions with the family. According to Mosenthal et al. [8] just using a checklist is not enough since the use of checklists for palliative processes requires a level of knowledge among the staff, and more evidence is needed to support these models [8, 38]. Continuity is required in order to achieve structure. In studies by Baggs et al. [15] and Nelson et al. [39] patients and their families felt a lack of continuity in their contact with health care professionals. After the implementation in this study the belief was that continuity increases a sense of security for all patients, and that patient-provider continuity also influences the team and the ability of health care professionals to maintain a palliative approach.

In a study by McConigley et al. [40] palliative knowledge was seen to change the attitudes of health care professionals and in a study by Friedrichsen et al. and Maxwell et al. [31, 41] such knowledge led to a greater understanding of palliative care. The results of this study revealed a belief that all patients benefit from the integration of a palliative approach into surgical care. However, there were also beliefs that these two types of care sometimes need to be separated since not all patients are palliative and the needs of staff, patients and their families may differ. Therefore a need for ability in one and same team to switch between different health care intentions.

Both before and after the implementation the participants believed that team collaboration regarding patients with palliative care needs was somewhat lacking. Problems with working in a group are usually due to a lack of appropriate methods of working and the dominance of traditions [34]. However, the implementation had created a group who regarded themselves as secure. This can be assumed to be a working group that is starting to develop into a team [34] and this aspect needs to be included in further studies of the implementation of palliative care.

According to Gott et al. [42] health care professionals working in either acute care or palliative care considered communication to be an important part of the decision-making regarding the transition to palliative care. Studies also show that there are different views regarding how collaboration [9, 15, 43] and communication [9, 44] function between the different professional groups in acute care with regard to decisions about palliative care. Before the implementation the belief regarding communication and relations between colleagues in this study was that they did not know much about each other’s work. They stayed mostly within their own professions and did not dare to give each other negative feedback. After the implementation participants described beliefs about a culture where they did not always dare to express opinions or disagreement, but also of beginning to be able to acknowledge the importance of their own professional role. According to Wheelan [34] a working group needs to have trust in each other’s competencies and the group needs to have a common ground and understanding; this is achieved by having an open discussion and trying to create an openness and an insight into each other’s work and the opinions of colleagues.

Ferrell et al. [45] argue that being able to discuss difficult subjects is one of the roles and responsibilities of health care professionals. The results of this study suggest that after the implementation there had been a change in the way delicate issues were communicated to patients due to participants believing that they had a greater understanding of their ability in this area, a deeper understanding concerning their ability to influence the discussion with a patient; however, it is not obvious in our data that a person-centred approach to communication with patients and their families [46] were adopted.

Earlier studies have shown that there is a lack of knowledge regarding symptom relief in patients with palliative care needs [39, 47–49] and that health care professionals would like more knowledge in this area [50] which the participants in this study also emphasised. The implementation resulted in increased knowledge regarding symptoms, with the main focus being on physical symptoms. According to Saunders [1] suffering does not only come from the body but it is also multidimensional. The results suggest that after implementation the participants had gained a broader perspective regarding various physical symptoms and were also starting to give attention to other dimensions. Beliefs that the attitudes of professionals may influence how patients talk about their symptoms were also seen. Specific interventions to relieve symptoms were not described either before or after the implementation.

The implementation initiated a change in the participants’ beliefs regarding the way that families behave. Benzein et al. [51] argue that health care professionals need to be aware of their own beliefs regarding the importance of the families. Health care professionals, patients and the families should be seen as equal participants in the care, where their expertise is given equal importance, and health care professionals should re-evaluate who is the expert and who has the preferential right of interpretation. According to Andershed [52] these days families are already caring for their terminally ill family member and the goal of health care professionals should be to become involved in the family’s situation, where the patient and their family are at the centre.

In a study by Nelson et al. [39] discussions with families were rated highly by both the families and the patients. The belief after the implementation in this study was that discussions with the family could save time, and that families appreciated being given information, even if it was negative. If families are made aware, this can be seen, according to Andershed [52] as being necessary for them to be able to deal with the situation and to give them a chance to prepare themselves for what is to come. Before the implementation families were offered the opportunity to talk with someone outside of the team that is someone who had not been involved in the care. After the implementation, the member of the team who was closest to the patient took more responsibility for discussions with the family. This can be seen as an attempt within the team to determine the needs that families have for information and to try to provide this at the right level and in a way they can understand [45].

Throughout the project the participants were given guidance in reflecting over their actions, which led to the development of knowledge about various beliefs concerning palliative care [19]. The project is believed to have resulted in an exchange of knowledge between colleagues and a feeling of wanting to share what they had learned with others [20]. It can be presumed that the implementation has resulted in reflective and professional practitioners [53]. To investigate what impact the implementation had on patients would require another study, but the results from this study can be seen as motivation to proceed with a future investigation of the effects on patients of using a combined consultation and integration strategy for the implementation of palliative care in hospitals.

### Method discussion

This study has weaknesses that need to be considered. The result is based on participants own accounts of beliefs, and one way to strengthen the design could be to utilize behaviour observation data. However, this would have required additional resources, and the chosen design was found appropriate given the limited knowledge related to implementation of palliative care in hospitals. The result, together with others [31, 35–37, 40, 41] contributes to a knowledgebase for providing explanations to processes of change in implementation of palliative care. Therefore the study was designed for a step-wise approach to the development of knowledge for feasible, effective, theoretically grounded and sustainable implementation strategies.

Other education programmes or changes in practice unknown to the researchers might have influenced the participants beliefs related to their practice. It can also be assumed that the team at the surgical department consisted of participants with an interest in palliative care and in learning new knowledge; they were probably also open to share their ideas and possibly to act as change agents. The differences between participants were reflected not only in their professions as surgeons and nurses, but also in the length of their professional experience. Participation by other professionals such as social workers, occupational therapists and nursing assistants would probably have resulted in different beliefs [29].

The mentor in focus groups plays an important role in encouraging interaction between the participants, and in stimulating discussions based on a variety of opinions; a role that can affect generation of data [29]. The mentor in the two first focus group discussions and the mentor in the last focus group discussion in this study used the same questions in all three focus group discussions. During analysis, differences were noted in where/how the two mentors chose to ask participants to relate/develop their responses. The mentors asked the questions in different ways and gave a different focus to the follow up questions. However, the implementation had influenced the group so that they dared to be more actively involved than before the implementation when they were more cautious.

Studies have shown that there can be difficulties with both collaboration [9, 15, 43] and communication [9, 44] within different professional groups in acute care. However, it is possible that results may have been different if the interviews had been carried out face to face instead of using focus groups. The participants in the study appeared at ease and they gave each other the opportunity to voice their comments. One idea of using focus groups was to capture the participants’ reasoning that would create a deeper understanding for each others comments. Given the teamwork approach suggested in palliative care it was thought that data could be obtained at a group level but there was also a resource issue which needed to be considered. The material was limited to three focus group discussions, but these still generated frequent descriptions of altered beliefs. Interpretation of the texts was performed with a focus on revealing beliefs rather than actual events. In an attempt to give the reader confidence and insight, quotes from participants are used where they are expressing themselves in their own words [30].

The processing of the data was, for practical reasons, carried out by a person who had not been present at the focus group discussions, and this may have produced a somewhat detached version of beliefs whereby implications may have been missed or misrepresented. However, considering the data generated as a text for analysis, it could also be considered a mean to ground the analysis only on data. Since the researchers had professional experience and specific expertise in palliative care yet very little experience in surgical care, this may also have influenced the analysis. The analysis led to instances when the material had to be put aside for a short time when the researchers own experience of palliative care triggered emotional reactions. Despite this, the beliefs discussed by the participants are reported here as faithfully as possible [30].

## Conclusion

This study confirms the feasibility of a palliative care implementation strategy with a combination of integration and consultation strategies and that beliefs regarding palliative care among professionals in surgical care can be changed through access to relevant knowledge so that health care professionals start to embrace palliative care as an integral and natural part of surgical care and treatment. The implementation can be said to have started a collective process of development among the participants. This process has resulted in a common palliative working method during contact with patients with palliative care needs and their families. Further, this micro level study contributes with suggesting specific contextual belief areas related to implementation of palliative care in hospitals, which add to the general beliefs described in implementation science: the importance of palliative care, working methods in palliative care, collegial support, discussions about diagnosis at the end of life, and the family of patients in palliative care.

Moreover, the change in beliefs involved differences regarding surgical care and palliative care. The fact that not all patients in a surgical department have needs of palliative care may create difficulties. The care on a surgical department requires an ability to switch between two different health care intentions i.e. goals and orientations of care.

Care at the end of life and the death of patients can occur in most health care departments implying that palliative care skills should be obvious part of team working methods irrespective of medical specialty and type of service. Different beliefs regarding what palliative care involves can affect whether and to what extent palliative care is included.

The implementation strategy that was evaluated using a combination of integration and consultation strategies can be seen as effective with regard to changing beliefs about palliative care. It should therefore in future studies be further tried and tested pertaining professionals’ behaviour and patient outcome in order to further develop appropriate and sustainable implementation strategies for palliative care.

## Additional file

Additional file 1: The participants’ and the palliative care specialists’ reflections on the implementation. (DOCX 12 kb)

## Abbreviation

PARiHS: Promoting Action on Research Implementation
