# Supplementary material for: Changes in professionals’ beliefs following a palliative care implementation programme at a surgical department: a qualitative evaluation
Source: BMC Palliat Care. 2017 Dec 28;16:77. doi: 10.1186/s12904-017-0262-4 (PMC5745985; doi:10.1186/s12904-017-0262-4)
Supplement: Additional file 1: — The participants’ and the palliative care specialists’ reflections on the implementation. (DOCX 12 kb) [file 12904_2017_262_MOESM1_ESM.docx]

**Additional file**

**Reflections on the implementation**

The opportunity to meet in groups and discuss in peace and quiet with the same people resulted in an exchange of knowledge between the surgeons and nurses, but also with other colleagues.

The opportunity for discussion led to new thoughts and ideas and the group dynamics acted as a catalyst for this development.

Regular meetings created a sense of security in the group, which resulted in the participants daring to relate their different thoughts and ideas, but also gave them an opportunity to ask questions.

It was seen as important that all the participants were present, and the implementation resulted in participants feeling much more involved and included.

Although the participants did not understand it at that time, the themed meetings gave them an opportunity to reflect with each other, a sort of debriefing.

Requests were made to be able to continue to meet regularly in the future, and to have themed meetings based on real cases, which could then be evaluated.

The implementation resulted in a change in the group dynamics. This went from the participants not interrupting each other and letting the mentors take a central role in leading the discussions, to them having lively discussions in which they interrupted each other with further questions. There was a genuine interest in getting to know more about each other's beliefs regarding palliative care and how this affected them.
